# Risk factors of postpartum stress urinary incontinence in primiparas: What should we care

**DOI:** 10.1097/MD.0000000000025796

**Published:** 2021-05-21

**Authors:** Jiejun Gao, Xinru Liu, Yan Zuo, Xiaocui Li

**Affiliations:** Key Laboratory of Birth Defects and Related Diseases of Women and Children (Sichuan University), Ministry of Education, Chengdu, China.

**Keywords:** care, management, nursing, primiparas, risk, stress urinary incontinence

## Abstract

Stress urinary incontinence (SUI) is a common clinical postpartum complication. It is necessary to explore the risk factors of postpartum SUI in primiparas to provide evidence support for preventing and reducing the occurrence of SUI.

Primiparas who were delivered in our hospital from March 2019 to October 2020 were identified, the personal information and related treatment details of SUI and no-SUI primiparas were collected and analyzed. Logistic regression analyses were conducted to identify the risk factors of postpartum SUI in primiparas.

A total of 612 primiparas were included, the incidence of SUI in primiparas was 32.03%. There were significant differences in the body mass index (BMI) before pregnancy, diabetes, abortion, delivery method, newborn's weight, epidural anesthesia, and duration of second stage of labor (all *P* < .05) between SUI and no-SUI group, and there were no significant differences in the age, BMI at admission, hypertension and hyperlipidemia SUI and no-SUI group (all *P* > .05). Logistic regression analyses indicated that BMI before pregnancy ≥24 kg/m^2^ (odds ratio [OR]: 2.109, 95% confidence interval [CI]: 1.042–4.394), diabetes (OR: 2.250, 95% CI: 1.891–3.544), abortion history (OR: 3.909, 95% CI: 1.187–5.739), vaginal delivery (OR: 2.262, 95% CI: 1.042–4.011), newborn's weight ≥3 kg (OR: 1.613, 95% CI: 1.095–2.316), epidural anesthesia (OR: 2.015, 95% CI: 1.226–3.372), and duration of second stage of labor ≥90 minutes (OR: 1.726, 95% CI: 1.084–2.147) were the risk factors of postpartum SUI in primiparas (all *P* < .05).

The clinical incidence of SUI in primiparas is relatively high. In clinical practice, medical staff should conduct individualized early screening for those risk factors, and take prevention measures to reduce the occurrence of SUI.

## 1 Introduction

Stress urinary incontinence (SUI) refers to the involuntary leakage of urine from the external opening of the urethra when abdominal pressure increases (such as sneezing, coughing, laughing, or exercising), which is a common gynecological urinary disease.^[1]^ It is reported that 25% to 55% of pregnant women have symptoms of urinary incontinence, and SUI occurs for the first time after vaginal delivery, accounting for 3.7% to 19%.^[2,3]^ Women who develop SUI after childbirth have higher bladder neck mobility before childbirth than those without urinary incontinence. In the 3 months postpartum, 34.3% of women have varying degrees of urinary incontinence, of which 3.3% have urine leakage or more frequently every day, and 8.5% of women need to wear pads.^[4]^ Therefore, the prevention and treatment of SUI is essential to the life quality of pregnant women.

The SUI of pregnant women has always been a clinical medical problem of concern. If the risk factors are not identified in the early pregnancy and the risk factors are not intervened as soon as possible, the late prognosis will be poor and the treatment cost will be high, which will bring a heavy burden to pregnant women, families, and society.^[5,6]^ There are many factors influencing the occurrence of SUI reported in previous studies, including body mass index (BMI), family history, pelvic floor muscle exercise, etc.^[7]^ However, existing studies^[8,9]^ mostly focus on postpartum or middle-aged and elderly urinary incontinence. The influencing factors of SUI in the population of primipara still need to be further explored. For this reason, it is necessary to retrospectively analyze the clinical data of primiparas, explore the risk factors that affect the occurrence of SUI during pregnancy, and provide a reference for formulating clinical SUI prevention and control strategies more reasonably, improving patient prognosis and reducing the incidence of urinary incontinence.

## 2 Methods

### 2.1 Ethical consideration

Our study had been verified and approved by the ethical committee of West China Second Hospital of Sichuan University (approval number: SD190023), and written informed consent had been obtained from all the included patients.

### 2.2 Population

This study selected pregnant women who were admitted to the delivery room in our hospital from March 2019 to October 2020 as study population, all the women at least stayed 3 days after delivery in our hospital.

The inclusion criteria were:

(1) Primiparous women;
(2) Pregnant women had a clear consciousness and were willing to participate in this study;
(3) Pregnant women had no history of kidney disease, or pelvic surgery.

The exclusion criteria for this study were:

(1) Pregnant women who had not undergone regular check-ups in our hospital;
(2) People with visual or hearing impairment;
(3) Pregnant women who were unwilling to participate in this study.

### 2.3 SUI diagnostic criteria

The SUI was diagnosed in comply with previous guidelines.^[10,11]^ According to the typical symptoms of SUI, that is, urine overflows when abdominal pressure increases in various degrees, such as laughing, coughing, sneezing or walking, and whether the urine flow stops immediately when the pressure is stopped.

### 2.4 Data collection

We collected personal information and related treatment details of included women, including age, BMI before pregnancy and at admission, diabetes, hypertension, hyperlipidemia, abortion history, delivery methods (vaginal delivery, cesarean section), newborn's weight, epidural anesthesia, and duration of second stage of labor.

### 2.5 Statistical methods

We use SPSS 23.0 to analyze the collected data. For those whose measurement data is normally distributed, the independent sample *t* test is used for those whose variances between groups are homogeneous. Count data and categorical variables were tested by chi-square test. The factors with *P* ≤ .10 in univariate analysis were further included in multivariate logistic regression analysis. All statistical tests are 2-sided probability tests. The difference was taken as statistically significant when *P* < .05.

## 3 Results

### 3.1 The characteristics of included primiparas

A total of 612 primiparas were included in this present study, and 196 primiparas had suffered from SUI, the incidence of SUI in primiparas was 32.03%. As showed in Table 1, there were significant differences in the BMI before pregnancy, diabetes, abortion, delivery method, newborn's weight, epidural anesthesia, and duration of second stage of labor (all *P* < .05) between SUI and no-SUI group, and there were no significant differences in the age, BMI at admission, hypertension, and hyperlipidemia SUI and no-SUI group (all *P* > .05).

**Table 1:** The characteristics of included primiparas (n = 612).

Variables	SUI group (n = 196)	Non-SUI group (n = 416)	*χ*^2^/*t*	*P*
Age (yr)	22.13 ± 5.25	23.09 ± 5.33	6.318	.058
BMI before pregnancy (kg/m^2^)	24.97 ± 4.54	21.22 ± 3.97	3.104	.034
BMI at admission (kg/m^2^)	27.12 ± 5.07	25.98 ± 6.22	3.138	.052
Diabetes	102 (52.04%)	62 (14.90%)	1.185	.007
Hypertension	44 (22.45%)	80 (19.23%)	1.227	.062
Hyperlipidemia	31 (15.82%)	58 (13.94%)	1.089	.101
Abortion history	84 (42.86%)	68 (16.35%)	1.124	.015
Delivery method				
Vaginal delivery	169 (86.22%)	313 (75.24%)	1.140	.031
Cesarean section	27 (13.78%)	103 (24.76%)		
Newborn's weight (kg)	3.51 ± 1.02	2.78 ± 1.04	1.701	.046
Epidural anesthesia	112 (57.14%)	108 (25.96%)	1.225	.013
Duration of second stage of labor (min)	103.19 ± 30.54	81.04 ± 32.71	9.196	.004

### 3.2 The risk factors of postpartum SUI in primiparas

We included the characteristics of included primiparas with significant difference into further logistic regression analyses. The variable assignment of multivariate logistic regression was presented Table 2.

**Table 2:** The variable assignment of multivariate logistic regression.

Factors	Variables	Assignment
SUI	Y	Yes = 1, no = 2
BMI before pregnancy (kg/m^2^)	X_1_	≥24 = 1, <24 = 2
Diabetes	X_2_	Yes = 1, No = 2
Abortion history	X_3_	Yes = 1, No = 2
Delivery method	X_4_	Vaginal delivery = 1, Cesarean section = 2
Newborn's weight (kg)	X_5_	≥3 = 1, <3 = 2
Epidural anesthesia	X_6_	Yes = 1, No = 2
Duration of second stage of labor (min)	X_7_	≥90 = 1, <90 = 2

As indicated in Table 3, The logistic regression analyses had found that the BMI before pregnancy ≥24 kg/m^2^ (odds ratio [OR]: 2.109, 95% confidence interval [CI]: 1.042–4.394), diabetes (OR: 2.250, 95% CI: 1.891–3.544), abortion history (OR: 3.909, 95% CI: 1.187–5.739), vaginal delivery (OR: 2.262, 95% CI: 1.042–4.011), newborn's weight ≥3 kg (OR: 1.613, 95%CI: 1.095–2.316), epidural anesthesia (OR: 2.015, 95% CI: 1.226–3.372), and duration of second stage of labor ≥90 minutes (OR: 1.726, 95% CI: 1.084–2.147) were the risk factors of postpartum SUI in primiparas (all *P* < .05).

**Table 3:** The logistic regression analysis on the risk factors of postpartum SUI in primiparas.

Variables	*β*	*S̄x*	OR	95% CI	*P*
BMI before pregnancy ≥24 kg/m^2^	0.126	0.217	2.109	1.042–4.394	.012
Diabetes	0.108	0.139	2.250	1.891–3.544	.028
Abortion history	0.113	0.121	3.909	1.187–5.739	.047
Vaginal delivery	0.142	0.107	2.262	1.042–4.011	.023
Newborn's weight ≥3 kg	0.112	0.104	1.613	1.095–2.316	.019
Epidural anesthesia	0.123	0.112	2.015	1.226–3.372	.041
Duration of second stage of labor ≥90 min	0.157	0.126	1.726	1.084–2.147	.017

## 4 Discussions

At present, there are relatively few studies on SUI of primiparas during pregnancy, and most of them are small sample studies, and its risk factors have not been uniformly recognized. Pregnancy and childbirth are directly related to the onset, development, and outcome of SUI.^[12]^ Previous studies^[13,14]^ have reported that pregnant women with urine leakage symptoms during pregnancy are more than twice as likely to have urine leakage again within 15 years after delivery than pregnant women without urine leakage symptoms during pregnancy. Studies^[15–17]^ have pointed out that urinary incontinence during pregnancy can double the risk of postpartum urinary incontinence, and primiparas without urinary incontinence before pregnancy are considered the best clinical model for normal pelvic tissue function. Therefore, we took primiparas as the research population. However, the risk factors of SUI during pregnancy are not yet fully clarified, clinically, the clinical experience of doctors is used to assess whether SUI is expected to occur in pregnant women, and there is a lack of objective evidence. The results of our study have found that the incidence of SUI in primiparas was 32.03%, and for patients with the BMI before pregnancy ≥24 kg/m^2^, diabetes, abortion history, vaginal delivery, newborn's weight ≥3 kg, epidural anesthesia, and duration of second stage of labor ≥90 minutes, early preventions and interventions are needed to reduce the occurrence of postpartum SUI.

The termination of pregnancy has marked the birth of a new life and the end of pregnancy, but the damage caused to women by pregnancy and childbirth has not end. Childbirth can cause damage to maternal pelvic floor function, and may cause pelvic organ prolapse and SUI.^[18]^ The incidence of postpartum SUI reported in previous literature is differed 7% to 40%, and the reason for the large difference in incidence may be related to the diagnosis standard of SUI, inspection time and delivery method, number of deliveries, ethnicity, and socioeconomic status.^[19,20]^ The pathogenesis of SUI is complicated, and there is no conclusion yet. It is generally believed that postpartum pelvic floor tissue neurological damage, myogenic changes or collagen tissue structure changes, leading to pelvic floor dysfunction and thus leading to the occurrence of SUI.^[21–23]^ The dysfunction of the urethral sphincter after childbirth, or atrophy of the urethral covering mucosa caused by other factors, urethral sphincter fibrosis, and pelvic nerve damage are also important pathological mechanisms of the onset of SUI.^[24,25]^

A study^[26]^ conducted follow-up visits to women and found that BMI before the first pregnancy was significantly related to the occurrence of urinary incontinence after delivery. At present, with changes in people's lifestyles and family members’ excessive attention to pregnant women, the phenomenon of overweight pregnant women is becoming more and more serious and common.^[27]^ Therefore, reasonable weight intervention and control should be carried out for the pregnant women population, and individualized nutrition and exercise guidance programs should be formulated for those women with higher BMI to reduce the incidence of SUI.^[28–30]^

Serati et al^[31]^ have conducted follow-up observations on 336 women with abnormal urination at 6 and 12 months postpartum, and have concluded that epidural anesthesia and the second stage of labor > 1 hour were significantly related to abnormal urination after delivery. It may be explained by that the size of the fetus's body weight needs to be considered in conjunction with the size of the mother's pelvis, if the pelvis is spacious, the damages to the pelvis are much less, the second stage of labor must be prolonged.^[32,33]^ At the same time, studies^[34,35]^ have reported that epidural anesthesia can lead to prolongation of the second stage of labor. Therefore, the control of fetus body weight, reduce the use of epidural anesthesia, and decrease the second stage of labor may be beneficial to reduce the occurrence of SUI.

Studies^[36,37]^ have pointed out that in young and middle-aged women, both vaginal delivery and cesarean section are more likely to develop SUI in the short term. SUI may also occur in primiparous women, but in older women, delivery methods no longer play an important role. The probability of SUI is similar between primipara and postpartum women. It is reported that 8/9 of the women in humans have given birth to offspring, most of which are vaginal delivery, but only 1/9 of them need surgery because of SUI or pelvic organ prolapse.^[38,39]^ For most women, vaginal delivery is not an inevitable cause of SUI. Obstetricians should not only consider that cesarean section can reduce the occurrence of postpartum SUI and arbitrarily increase cesarean section without obstetric indication.^[40,41]^ It is worth noting that vaginal delivery is still the most important and healthy way of delivery. It is recommended that pregnant women have a reasonable diet, appropriate activities, control weight gain during pregnancy, pay attention to the repair of perineum injury during childbirth, and examine the postpartum pelvic floor muscle tissue and the pelvic floor.^[42–44]^ Besides, correct assessment and improvement of deep and shallow muscle function is an important means to prevent and treat SUI.^[45]^

Several limitations in this present study must be concerned. First, we did not calculate the sample size, and we did not conduct preventive power analyses on the identified risk factors, it may be unclear about the preventive power of our identified risk factors, future studies with larger sample size are needed to elucidate the preventive power of related risk factors. Second, since our study is retrospective analysis, many laboratory results could not be included for data analysis since some of included patients missing the related data, the possible errors in properly diagnosing incontinence should be concerned, future studies with rigorous prospective design are needed.

## 5 Conclusions

In summary, we have found that the SUI in primiparas is very common, and BMI before pregnancy ≥24 kg/m^2^, diabetes, abortion history, vaginal delivery, newborn's weight ≥3 kg, epidural anesthesia, and duration of second stage of labor ≥90 minutes were risk factors for postpartum SUI. With the progress of society and the needs of national health, early screening and prevention of urinary incontinence have become an inevitable trend to improve the quality of life. After identifying the risk factors of SUI in pregnant women, what kind of intervention measures should be adopted to individualize pregnant women and ultimately reduce the incidence of SUI or improve the symptom tolerance of pregnant women needs further exploration. However, the sample size of this study is small and the follow-up time is short. Therefore, the results of the study should be treated with cautions, and the risk factors and interventions of SUI still need to be further explored in future large-sample and high-quality studies.

## Author contributions

**Conceptualization:** Xinru Liu, Yan Zuo, Xiaocui Li.

**Data curation:** Jiejun Gao, Xinru Liu, Xiaocui Li.

**Formal analysis:** Jiejun Gao, Xinru Liu, Yan Zuo, Xiaocui Li.

**Investigation:** Jiejun Gao, Xinru Liu, Yan Zuo, Xiaocui Li.

**Methodology:** Jiejun Gao, Yan Zuo.

**Project administration:** Jiejun Gao, Yan Zuo.

**Resources:** Xinru Liu, Yan Zuo.

**Software:** Jiejun Gao, Xinru Liu.

**Supervision:** Jiejun Gao.

**Visualization:** Jiejun Gao.

**Writing – original draft:** Jiejun Gao.

**Writing – review & editing:** Xinru Liu.
